# Mechanotransduction in skin wound healing and scar formation: Potential therapeutic targets for controlling hypertrophic scarring

**DOI:** 10.3389/fimmu.2022.1028410

**Published:** 2022-10-17

**Authors:** Jiayi Yin, Shiming Zhang, Chao Yang, Yan Wang, Bing Shi, Qian Zheng, Ni Zeng, Hanyao Huang

**Affiliations:** State Key Laboratory of Oral Diseases and National Clinical Research Center for Oral Diseases and Department of Oral and Maxillofacial Surgery, West China Hospital of Stomatology, Sichuan University, Chengdu, China

**Keywords:** mechanotransduction, hypertrophic scar (HTS), wound healing, mechanical forces, signaling/signaling pathways

## Abstract

Hypertrophic scarring (HTS) is a major source of morbidity after cutaneous injury. Recent studies indicate that mechanical force significantly impacts wound healing and skin regeneration which opens up a new direction to combat scarring. Hence, a thorough understanding of the underlying mechanisms is essential in the development of efficacious scar therapeutics. This review provides an overview of the current understanding of the mechanotransduction signaling pathways in scar formation and some strategies that offload mechanical forces in the wounded region for scar prevention and treatment.

## Introduction

The skin is the largest organ of the human body which forms a protective barrier between the body and the external environment. After injury, the process of wound healing immediately starts to maintain the integrity of the skin. In adult humans, the ideal healing outcome is skin regeneration, which refers to the return of the injured site to the pre-wounding form with the recovery of secondary skin elements, ultrastructure and mechanical properties. However, injured skin generally heals by fibrotic scar tissue with permanent defective structure and impaired functions (1), and sometimes develops hypertrophic scarring (HTS). HTS causes a bad appearance to the patients when happening to the face, arms or legs, which sometimes impacts the patient’s quality of life, and current therapies cannot efficiently attain scar-less healing or reverse fibrosis.

Recent studies shed light on the effect of mechanical forces in HTS formation, and the mechanotransduction signaling pathway can be a potential target to reduce scarring and promote skin regeneration. In this review, we aim to provide an overview of the mechanotransduction signaling pathways in HTS formation. We also discuss possible strategies that can offload mechanical forces in the wounded region for treating and preventing HTS.

## Classic stages of adult cutaneous wound healing

Wound healing in humans is an important but complicated process, which consists of four overlapping but distinct stages: hemostasis, inflammation, proliferation and remodeling (2). A multitude of cells and mediators play specialized roles in this process.

### Hemostasis

The first response, hemostasis, occurs immediately after an injury to the skin. The ruptured vessels constrict by reflexive contracture of the vascular smooth muscle, mediated by vasoconstrictors such as endothelin (3, 4). The subendothelial matrix proteins are exposed to the blood components, leading to the tethering of platelets at the injured site (5–7). The activated platelets release numerous mediators like platelet-derived growth factor (PDGF), transforming growth factor-β (TGF-β), epidermal growth factors (EGF) and basic fibroblast growth factor (bFGF) (8, 9). Aggregated platelets provide the surface for activation of coagulation complexes (10). The classic coagulation cascade consists of two converging pathways: extrinsic and intrinsic. With a distinct trigger, they both lead to factor X activation, followed by conversion of prothrombin to thrombin. Thrombin can convert fibrinogen into fibrin, which forms a crossed-link mesh that further stabilizes the growing platelet plug and form the matrix as a scaffold for the infiltration of other cells (6, 10–12).

### Inflammation

The establishment of vasoconstriction lasts a few minutes, taken over by vasodilation with increased permeability, allowing the extravascular migration of inflammatory cells.

Neutrophils are the first subset of leukocytes to enter the injured area (13). They dominate the inflammatory phase within 24 to 48 hours post-wounding, with macrophages taking over from approximately 36 hours (14, 15). The first signal of neutrophil recruitment is thought to be damage-associated molecular patterns (DAMPs), such as DNA, histones, high mobility group protein B1 (HMGB1), and adenosine triphosphate (ATP) (16, 17). In the case of infection, neutrophils also rapidly detect pathogen-associated molecular patterns (PAMPs) such as lipopolysaccharide, (LPS) (16, 18). The long-term chemoattractants mainly comprise chemokines and lipid mediators such as C-X-C motif chemokine ligand 8 (CXCL8) and leukotriene B_4_ (LTB_4_) (17, 19, 20). Neutrophils are armed with various antimicrobial substances and proteases, which can be released into phagolysosomes after phagocytizing microbe or released into extracellular space (13, 20, 21). Besides, neutrophils generate reactive oxygen species (ROS), form neutrophil extracellular traps (NETs), and release various cytokines and chemotaxins to modulate the inflammatory phase (22). After successful response to the injury, neutrophils undergo apoptosis and NETosis or leave the site of tissue damage in a process termed neutrophil reverse migration (17, 23).

Followed by the influx of neutrophils, monocytes are recruited to the wound site as responders to DAMPs and PAMPs, which further differentiate into the macrophages (15, 24). During early wound healing, macrophages exhibit pro-inflammatory phenotype, described as M1-like macrophages (25). M1-like macrophages have a high phagocytic capacity. They can remove dead cells (e.g., apoptotic neutrophils), cellular debris, bacteria and many others (26). Meanwhile, they release antibacterial mediators and secrete inflammatory cytokines such as tumor necrosis factor (TNF)-α, interleukin (IL)-6, IL-1β, monocyte chemoattractant protein-1 (MCP-1), etc. to attract defense components and amplify the inflammatory state (26–28). They also synthesize MMPs to break down the extracellular matrix (ECM) to aid in cell infiltration (26, 29). In the late inflammatory stage, the activated M1-like phenotype gradually skews toward an anti-inflammatory cell type called the M2-like macrophage (25, 28). The M2-like macrophage dampens inflammation by expressing anti-inflammatory cytokines (e.g., IL-10) and growth factors (e.g., PDGF, TGF-β, VEGF) (26, 29, 30). Those signals contribute to new vessels formation, encourage the migration of keratinocytes, fibroblasts, and endothelial precursor cells, induce myofibroblasts transition (10, 31). Collectively, M2-like macrophage play important role in the late stage of inflammation and is highly involved in the proliferation phase.

### Proliferation

The proliferative phase is achieved through three main steps: re-epithelialization, angiogenesis and the formation of granulation tissue (32). The first event is that keratinocytes at the wound edges proliferate actively and migrate to the denuded wound surface, establishing coverage of the wound bed (1, 33). In this progress, the epithelial-to-mesenchymal transition (EMT) occurs where the epithelial cells differentiate into novel fibroblast-like cells, lose intercellular junctions and shift into a dynamic state (34, 35). The migration ceases and gradually stops when the keratinocytes re-constructed dermo-epidermal junctions (35, 36).

The restoration of the vascular system is initiated by growth factors such as TGF-β, PDGF, bFGF secreted by palates, M2-like macrophages, endothelial cells etc (37, 38). Many of the newly formed vessels are leaky or not functional, and most vessels regress through cell apoptosis until the density of blood vessels returns to that of pre-wounding skin (38–40). The vessels mature into arteries and venules by recruitment of smooth muscle cells in the form of pericytes (33, 41).

Concurrently, the fibroblasts activated by growth factors migrate into the site of injury (35). The fibroblasts associated with macrophages, type III collagen and the sprouts of capillaries replace the fibrin matrix with granulation tissue (1, 36). During granulation formation, the myofibroblasts become abundant, which are derived from various sources such as fibroblasts, epithelial cells through EMT and mesenchymal stem cells (42, 43). Both fibroblasts and myofibroblasts lay down disorganized ECM composed of collagen (mainly type III), fibronectin, hyaluronic acid, and proteoglycans (1). Besides, myofibroblasts synthesize α-smooth muscle actin (α-SMA) and generate contractile forces which facilitate wound closure (43).

### Remodeling

Most cells involved in the healing process undergo apoptosis or exit from the wound in this phase. Remodeling of the ECM spans the entire injury response, beginning with the formation of fibrin clot, and ending years later with the formation of a mature scar (44). The ECM gradually shifts into a denser one primarily composed of type I collagen (45, 46). The well-balanced collagen degradation and synthesis are mainly mediated by MMPs secreted by anti-inflammatory macrophages, fibroblasts and keratinocytes (44). Meanwhile, the structure of ECM is aligned to densely arranged collagen bundles that are oriented parallel to the wound surface, enhancing the stiffness of the scar (46).

## Mechanical forces and HTS formation

The preferred scar after wound healing is narrow, flat, and soft, with similar color and texture to the adjacent skin. Conversely, aberrant wound healing results in unfavorable scars. HTS is a fibroproliferative disorder that may arise after deep cutaneous injuries caused by trauma, burns, surgery, etc (47). HTS is characterized by densely packed type III collagen fibers parallel to the epidermal surface and a lack of cutaneous appendages, leading to functional deficits and poor aesthetic outcomes (48). Previous studies have established that the mechanical environment at the wound site is strongly correlated with HTS formation. For example, it is a clinical observation that regions where the skin is often under tension and wounds with soft tissue loss are more prone to develop HTS, such as injuries at joints, lower abdomen, sternum and cleft lip scars, jagged wounds, etc (49). Incisions lying perpendicular to Langer’s skin tension lines show a higher incidence of HTS formation, while those parallel to Langer’s lines tend to heal with less scarring (50). Besides, shielding dermal wounds from mechanical stresses with tension-reducing strategies has been shown to mitigate scar formation (51). Those highlight the importance of understanding the molecular mechanisms and mechanical forces during wound healing and scar formation.

**Figure 1:**
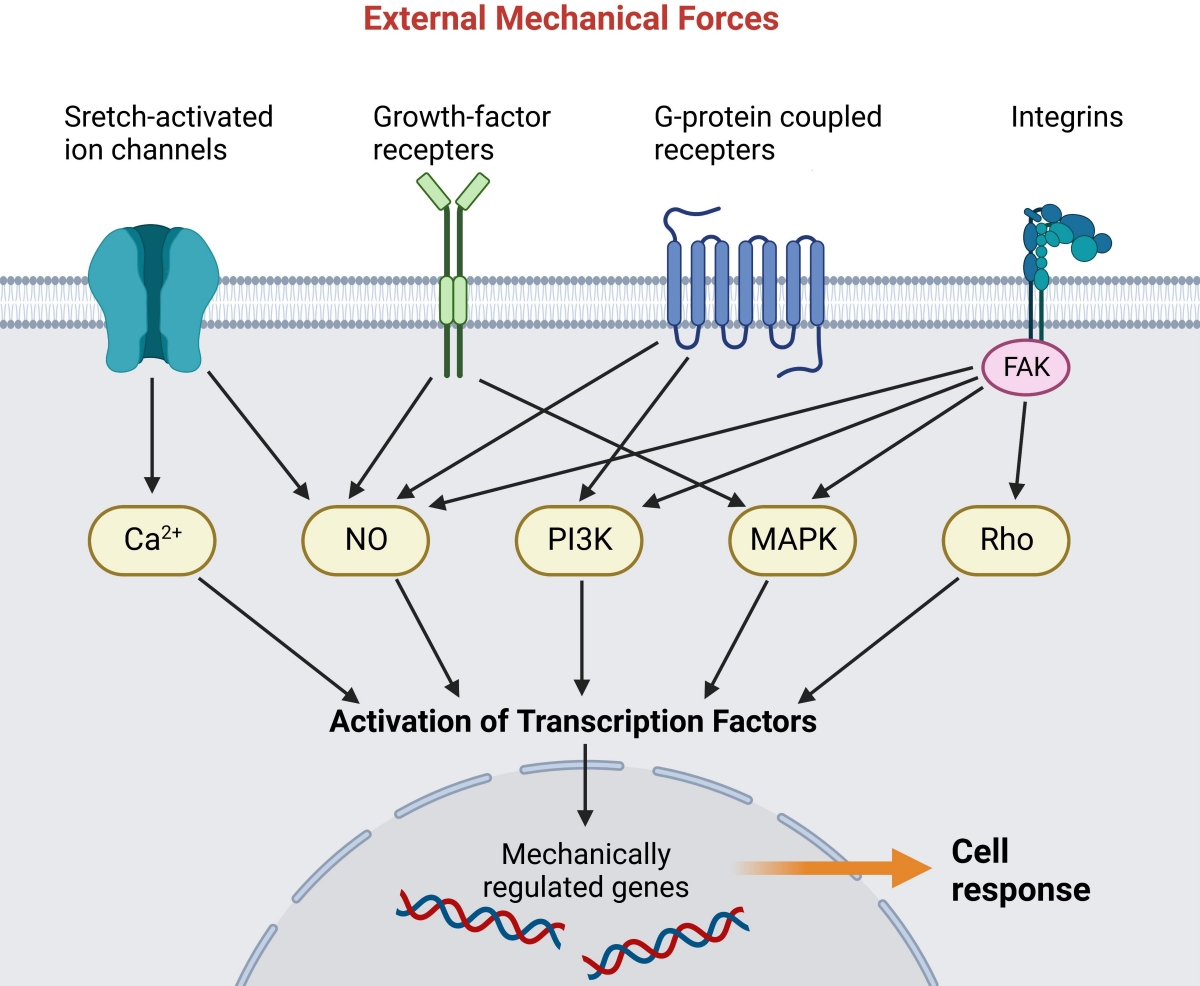
Mechanoreceptors transmit external mechanical forces and activate various intracellular signaling pathways. The signal cascades activate transcription factors that translocate into the nucleus and activate mechanically regulated genes (55). (Created with BioRender.com).

Mechanotransduction refers to the process that cells transmit mechanical forces to intracellular biochemical signals (52, 53). Cells perceive extrinsic mechanical cues through diverse mechanosensitive proteins (referred to as mechanosensing), with cadherins and integrins being the most common mechanical signaling interfaces (54). Then the signal propagation is activated, enabling cells to respond to mechanical stimuli by altering their biological capabilities (**Figure 1**) (56). The cellular response largely depends on the highly dynamic cytoskeleton, which is generated by the mechanical properties and interactions among actin filaments, microtubules, intermediate fibers, etc (57). Mounting studies indicate improper mechanotransduction can elicit pathological wound healing, including overhealing (fibrosis, keloids) and underhealing (chronic wounds) (52, 58). Nevertheless, the specific components and pathways involved in mechanotransduction still need to be elucidated. Here we introduce some mechanotransduction signaling pathways that have been shown to play crucial roles in wound healing and stretch-induced HTS formation.

### Integrins-FAK signaling

**Figure 2:**
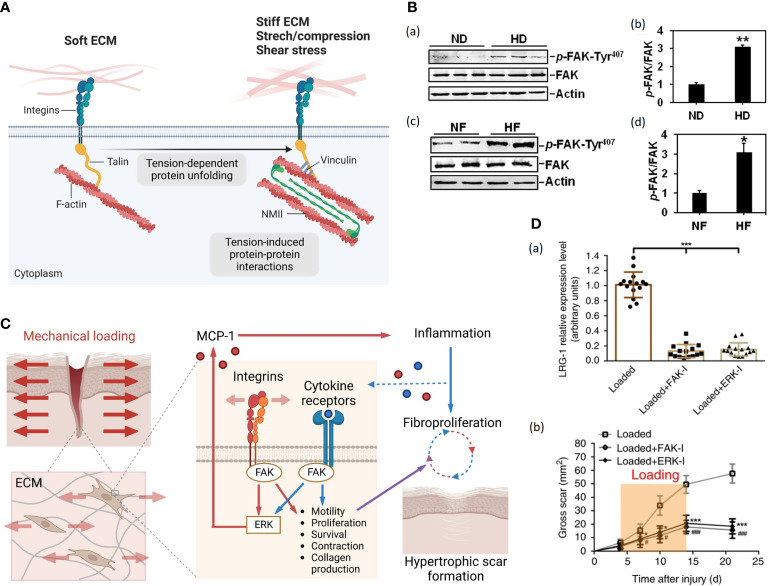
**(A)**. Cells attach to the ECM through integrins, which couple to the cytoskeleton *via* several F-actin binding proteins, here depicted for simplicity by the talin protein (56). On soft ECM, in the absence of resisting forces and of opposing cytoskeletal tension, talin remains in a closed conformation, limiting the maturation of focal adhesions (FAs) (left) (56). On stiff ECM or in the presence of mechanical forces, higher contractile forces generated by non-muscle myosin II (NMII) activity lead to talin unfolding and recruitment of FAs, depicted here by vinculin, initiating signaling within the cell (right) (56). (Created with BioRender.com). **(B)** (a) Immunoblots show the expression of p-FAK-Tyr407 and FAK in lysates from normal skin dermis (ND) and hypertrophic scar dermis (HD) (59). Actin serves as an equal loading control. (b) Bar graphs show that the relative protein level of p-FAK-Tyr407 is significantly up-regulated in HD compared to that in ND (59). (c) Immunoblots show the expression of p-FAK-Tyr407 and FAK in lysates from normal skin fibroblasts (NF) and hypertrophic scar fibroblasts (HF) (59). Actin serves as an equal loading control (59). (d) Bar graphs show that the relative protein level of p-FAK-Tyr407 is up-regulated in HF compared to that in ND (59). **(C)** Schematic of the proposed vicious cycle of HTS driven by mechanical activation of local and systemic fibroproliferative pathways through fibroblast FAK (60). (Created with BioRender.com). **(D)**. FAK or ERK inhibitor injection (a) blocks mechanical loading-induced LRG-1 expression and (b) attenuates scar formation (61).

Focal adhesions (FAs) are macromolecular assemblies that consist of integrins and an extensive array of adaptor proteins. The integrins, comprising heterodimers of α and β subunits, form a mechanical linkage and construct two-way communication between ECM and actin cytoskeleton (**Figure 2A**) (62, 63). Focal adhesion kinase (FAK) is a non-receptor cytoplasmic tyrosine kinase that localizes to FAs and transduces signals from them (62). After cutaneous injury, FAK is activated, and this process is potentiated by mechanical loading through phosphorylation (60, 64). However, the detailed cascades in HTS formation remain unclear. Su et al. found that the protein level of p-FAK-Tyr (the phosphorylated form of FAK) was significantly up-regulated in human HTS dermis *in vivo* and human HTS-derived fibroblast *in vitro* (**Figure 2B**) (59). Wong et al. constructed a murine HTS model and found that fibroblast-specific deletion of FAK resulted in less fibrosis with reduced numbers of myofibroblasts positive for α-SMA. Besides, application of strain to human fibroblasts *in vitro* demonstrated that FAK acted through extracellular-related kinase (ERK) to mechanically trigger the secretion of MCP-1, a potent chemokine linked to human fibrosis, and inhibition of either FAK or ERK blocked strain-induced MCP-1 secretion, indicating that mechanical force regulated pathologic scar formation through inflammatory FAK-ERK-MCP-1 pathways (**Figure 2C**) (60). Besides, therapies targeting FAK have shown an anti-scarring effect. Wong et al. administrated PF573228 (a FAK inhibitor) daily to wounds in the murine HTS model, generating less scar formation (60). Ma et al. developed pullulan-collagen-based hydrogels with controlled delivery of FAK inhibitor (FAKI) (65). Applying these FAKI to murine HTS model for down-regulating α-SMA expression in full-thickness excisional and burn wounds with accelerated wound closure, decreased scar formation and increased mechanical properties (65). Gao et al. found that mechanical force-induced-FAK/ERK axis activation promote leucine-rich-alpha-2-glycoprotein 1 (LRG-1) expression through ELK1 transcription factor, resulting in pathological angiogenesis and HTS formation (61). Injection of FAK or ERK inhibitor to mechanical load-induced mouse HTS tissue exhibited attenuated scar formation with decreased LRG-1expression (61) (**Figure 2D**).

Nevertheless, Jasnuszyk et al. reported that keratinocyte FAK-deleted mice displayed delayed wound healing with reduced collagen density and dermal thickness, which is caused by over-activation of MMP9 after FAK knockout (58). Those studies suggest a complex effect of FAK on wound repair; only with a certain extent of activation could the signaling pathway lead to proper wound closure.

### Wnt/β-catenin signaling

**Figure 3:**
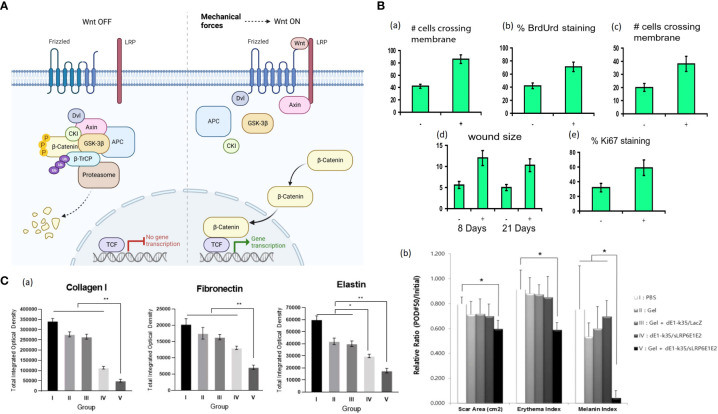
**(A)** Wnt/β-Catenin Signaling in Cells. Without the Wnt signal, the destruction complex of Axin, APC, the Ser/Thr kinases GSK-3 and CK1 and β-TrCP is formed in the cytoplasm. The complex specifies a β-TrCP recognition site on β-catenin and phosphorylates β-catenin by CK1 and GSK-3. After phosphorylation and ubiquitination, β-catenin is degraded by the proteasome. The binding of Wnt to the Frizzled/LRP receptor complex induces the dissociation of DC. The stabilized β-catenin proteins accumulate in the cytoplasm and translocate into the nucleus, subsequently binding TCFs and activating transcription. Mechanical force is associated with Wnt/β-Catenin Signaling, but further investigation is needed to demonstrate the correlation (68, 69). (Created with BioRender.com). **(B)** (a) Motility increases with induction of stabilized β-catenin as measured by the number of cells per high-powered field crossing the membrane using a modified Boyden chamber (70). (b) Proliferation significantly increases with transgene induction as measured by using the percentage of cells incorporating BrdUrd (70). (c) Cell invasiveness is increased after transgene induction as determined by the number of cells per high-power field crossing Matrigel (70). (d) Wounds are more significant in animals expressing the stabilized β-catenin (70). (e) Ki-67 staining is present in a higher percentage of cells in β-catenin-overexpressing mouse wounds compared to untreated wounds, indicating an increased proliferation rate (70). +, treated with doxycycline; −, not treated. **(C)** (a) Representative sections of tissues from the central areas of the scar 50 days after treatment were stained, and expression levels of type I collagen, elastin, and fibronectin were semi-quantitatively analyzed with MetaMorph^®^ (71). (b) The sLRP6E1E2-expressing Ad plays a prominent role in the reduction of scar surface area and color (mainly composed of erythema and melanin) (71). Ad with alginate gel-matrix system showed more effective biologic activities than naked Ad (71). *P < 0.05; **P < 0.01.

In non-regenerating vertebrates, including mammals, the Wnt/β-catenin pathway activity is maintained only in specific organs/tissues with high cell turnover, such as intestinal epithelium, epidermis, etc (66). The hallmark of Wnt/β-catenin signaling is the accumulation and translocation of β-catenin into the nucleus (67). The stability of β-catenin is regulated by the formation of a destruction complex (DC) consisting of Axin, adenomatous polyposis coli (APC), glycogen synthase kinase-3 (GSK-3), casein kinase 1 (CK1), protein phosphatase 2A (PP2A) and the E3-ubiquitin ligase β-TrCP (68). In the absence of Wnt signal, cytoplasmic β-catenin is hyperphosphorylated within the DC and then undergoes ubiquitination and degradation (67, 69). With stimuli like mechanical loading, Wnt binds to its receptors, FZD and LRP heterodimers, the downstream signal mediators, such as Disheveled (DVL) and Axin, are relocalized to the receptors complex on the cell membrane, and the DC is dissociated. As a result, free β-catenin rapidly accumulates in the cytoplasm and translocates into the nucleus, where it binds with the T cell factors (TCFs) for target gene expression (**Figure 3A**) (68, 69). Without the Wnt signal, the destruction complex of Axin, APC, the Ser/Thr kinases GSK-3 and CK1 and β-TrCP is formed in the cytoplasm. The complex specifies a β-TrCP recognition site on β-catenin and phosphorylates β-catenin by CK1 and GSK-3. After phosphorylation and ubiquitination, β-catenin is degraded by the proteasome. The binding of Wnt to the Frizzled/LRP receptor complex induces the dissociation of DC. The stabilized β-catenin proteins accumulate in the cytoplasm and translocate into the nucleus, subsequently binding TCFs and activating transcription (**Figure 3A**).

Like the integrins-FAK signaling, the Wnt/β-catenin signaling in cutaneous injury response displays a dichotomy of regeneration and fibrosis (66). Amini-Nik et al. demonstrated that mice lacking β-catenin in macrophages showed defected cutaneous wound repair and the macrophages lacking β-catenin were impaired in their ability to migrate, adhere to fibroblasts and produce TGF-β1 (72). Cheon et al. generated a mouse model in which stabilized β-catenin was expressed in mesenchymal cells under the control of a tetracycline-regulated promoter and found that β-catenin transcriptional activity in wound fibroblasts is upregulated, causing hyperproliferation, high motility and increased invasiveness of fibroblasts and cutaneous scars with larger sizes and increased amounts of collagen (**Figure 3B**) (70). Similarly, dermal mesenchymal cells in human HTS displayed a prolonged duration of elevated β-catenin protein and increased expressions of MMP7 and fibronectin (73). Besides, there has been shown evidence of cross-talk between Wnt/β-catenin and TGF-β pathways. For instance, Wnt signaling can up-regulate the expression of TGF-β, and TGF-β1 can promote β-catenin signaling (74, 75). Yang et al. applied sodium alginate-based hydrogel loaded with decoy Wnt receptor (sLRP6E1E2)-expressing adenovirus (Ad) to the pig scar model (71). The Ad/gel reduced collagen I, elastin, fibronectin and TGF-β1 mRNA expression and up-regulated TGF-β3 mRNA expression, ultimately yielding scar with decreased size and color (**Figure 3C**) (71).

### YAP/TAZ

**Figure 4:**
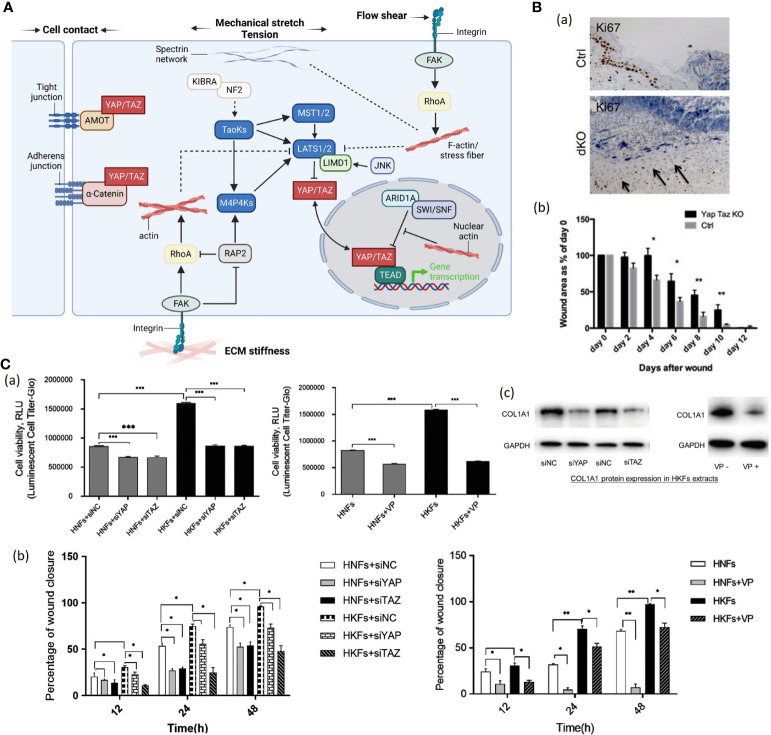
**(A)**. The schematic diagram YAP/TAZ signaling. Mechanical cues control YAP/TAZ activity through both Hippo-dependent and -independent pathways (78). (1) In response to cell-cell contact, AMOT directly binds to YAP and thus sequesters YAP at tight junctions (78). Adherens junction protein E-cadherin trans-dimerize and subsequently inactivate YAP/TAZ through the MST1/2-LATS1/2 kinase cascade (78). (2) ECM stiffness transducted *via* Integrin-FAK signaling promotes actin polymerization and stress fiber formation, subsequently inactivating the Hippo kinase cascade (78). Polymerized nuclear actin binds to ARID1A-SWI/SNF complex, relieving its sequestration of YAP/TAZ (78). Stiffness-regulated GTPase RAP2 activates MAP4Ks and inhibits Rho GTPases, inducing inhibitory action of LATS1 and LATS2 kinases on cytoplasmic YAP/TAZ (78). Stiffness-activated JNK phosphorylates LIMD1, which binds to LATS1/2, and activates YAP/TAZ (78). (3) Mechanical stretch or tension modulates YAP/TZ activities through actin cytoskeleton as well (78). Spectrin, a cytoskeletal protein, plays a crucial role in connecting the cellular tension-sensing system to the Hippo regulation network (78). (4) Flow shear patterns and speeds regulate the Hippo kinase cascade activity in endothelial cells *via* an integrin–Gα12/13–RhoA axis, respectively (78).(Created with BioRender.com). **(B)** (a) Proliferation of cells marked by Ki67 staining is reduced in dKO wounds versus control animals. Values are means ± s.e.m. *P<0.05, **P<0.01 (79). (b) Quantification of wound healing rates at each stage in control versus dKO animals (79). **(C)** (a) (Left) Human keloid fibroblasts (HKFs) had increased metabolic activity demonstrated by high RLU value than human normal fibroblasts (HNFs) (n = 3) (80).(Right) Knockdown of YAP or TAZ and verteporfin treatment decreased cell metabolic activity in both HNFs and HKFs (n = 3) (80). (b) (Left) Quantification of cell migration from 3 separate experiments after knockdown of YAP or TAZ in both HNFs and HKFs (80). (Right) Quantifying cell migration from 3 separate experiments to test verteporfin’s effect on HNF and HKF migration (80). VP, verteporfin; siNC, siRNA control; HNFs, human normal fibroblasts; HKFs, human keloid fibroblasts; *p < 0.05; **p < 0.01 (c) (Left) Western blot shows reduced protein expression of COL1A1 in HKFs after siRNA interference (80). (Right) Western blot shows COL1A1 protein expression in HKFs without verteporfin (VP-) and with verteporfin (VP+) treatment (80). ***P < 0.001.

Yes-associated protein (YAP) and transcriptional coactivator with PDZ-binding motif (TAZ) are two related coactivators that contribute to mechanical signal transduction (76). YAP and TAZ are regulated by multiple inputs, including Hippo kinase cascade, Wnt signaling, G-protein coupled receptors (GPCR) and mechanical force (77). In the presence of extracellular mechanical cues (e.g., ECM stiffness, cell attachment or detachment, cellular tension), YAP/TAZ translocates to the nucleus with DNA-binding transcription factors such as TEADs, promoting cell proliferation and inhibiting differentiation, whereas the absence of mechanical forces leads to YAP/TAZ nuclear export (**Figure 4A**) (78, 81–83). The precise mechanisms of this YAP/TAZ nucleocytoplasmic shuttling remain elusive. Those features suggest that YAP/TAZ are not only sensors of the mechanical cue but also active mediators of cell metabolic functions.

Nuclear localization of YAP and TAZ was particularly enriched in basal layer cells of the interfollicular epidermis and the hair follicle in human (79, 84). Elbediwy et al. generated YAP/TAZ double conditional knockout (dKO) mice and found that YAP/TAZ dKO mice showed decreased cell proliferation at the wound site and delayed wound closure (**Figure 4B**) (79). Lee et al. constructed a mouse skin wound model and detected enhanced nuclear localization of YAP and TAZ in the injured dermis (84). Besides, conditional YAP/TAZ knockout in the dermis or application of interfering RNAs to the wound site leads to delayed wound closure due to decreased cell proliferation, indicating that YAP/TAZ promotes wound healing (79). Gao et al. found that human keloid fibroblasts have higher YAP/TAZ mRNA and protein levels than normal skin tissue on primary culture (80). Additionally, knockdown of YAP/TAZ with siRNA interference technique or usage of verteporfin significantly reduced proliferation, migration, survival and collagen production of human keloid fibroblasts (**Figure 4C**), indicating that YAP/TAZ inhibitor had potential clinical significance for HTS management (80).

### PI3K/Akt signaling

The phosphatidylinositol 3-kinase (PI3K)/Akt signaling is involved in a broad range of cellular regulatory processes, including cell proliferation, metabolism, motility, and secretion (85). Additionally, it is responsible for maintaining skin homeostasis. In cutaneous injury, up-regulation of phosphorylated Akt was observed in the wounded site (86). Paterno et al. constructed a mouse wound model and found that dermal fibroblasts of mechanically loaded incisions exhibited more robust Akt activation than those of unloaded wounds (87), which demonstrated the correlation between mechanical force and PI3K/Akt signaling.

**Figure 5:**
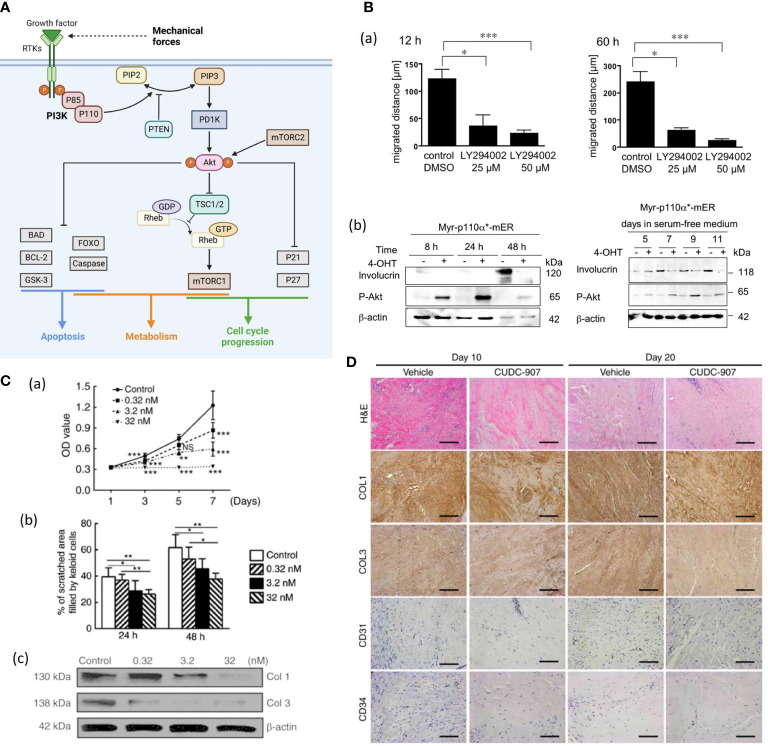
**(A)**. Schematic illustration of the PI3K/Akt/mTOR signaling pathway. The activation of receptor tyrosine kinases (RTK) activates phosphatidylinositol 3-kinase (PI3K), which in turn phosphorylated PIP2 to PIP3 (89). While phosphatase and tensin homolog (PTEN) can dephosphorylate PIP3 (89). Then Akt is recruited to the plasma membrane and phosphorylated by mTORC2 (89, 90). Akt regulates several cellular processes through a variety of downstream proteins like glycogen synthase kinase 3-beta (GSK-3β), Forkhead Box O (FOXO) etc. (89) Besides, AKT can phosphorylate and inactivate RAS homolog enriched in brain (Rheb) and retention of the Rheb-GTP activates mTORC1 (89). Mechanical force shows association with Akt expression, but further investigation is needed to demonstrate the correlation (87). (Created with BioRender.com). **(B)** (a) Treatment with LY294002 (a PI3K inhibitor) markedly reduces the motility of human keratinocytes (91). (b) Expression of the differentiatuin marker involucrin increases in solvent-treated Myr-p110α*-mER cells (91). The 40OHT-treated Myr-p110α*-mER cells failed to induce involucrin protein expression under these conditions (91). **(C)** (a) Cell Counting Kit-8 analysis revealed that cell proliferation was inhibited by CUDC-907 at different concentrations, with significant differences among treatment groups (92). (b) Semi-quantitative analysis of the scratch assay results (n=20) (92). (c) CUDC-907 treatment decreased the production of COL1 and COL3 at 72 h post-treatment, as demonstrated by western blot analysis (92) (*p<0.05, **p<0.01 and ***p<0.001). **(D)** Hematoxylin and eosin, and immunohistochemical staining revealed fewer cell numbers, decreased deposition of COL1 and COL3, and fewer formed microvessels (n=15) (92). Magnification, ×200; scale bar=250 μm. Data are presented as the mean ± standard deviation of the mean. COL1, type I collagen; COL3, type III collagen; CD31, platelet endothelial cell adhesion molecule; CD34, hematopoietic progenitor cell antigen CD34 (92). NS, no significance.

Human cells express three classes of PI3Ks, of which Class I is the most widely investigated. It is a heterodimer composed of a catalytic subunit and a regulatory subunit (88). The serine/threonine kinase Akt is a proto-oncogene. The activation of Akt occurs through multiple upstream pathways including PI3K (85). The phosphatidylinositol triphosphate (PIP3), transformed from PIP2 by the stimulus of PI3K, can activate Akt cascades, followed by Akt translocating from cytoplasm to the plasma membrane and phosphorylating its downstream targets, regulating numerous cell activities such as proliferation, metabolism, apoptosis, transcription, and protein synthesis (**Figure 5A**) (88, 89, 93).

Pankow et al. generated HaCaT keratinocyte cell lines stably expressing a 4-OHT-inducible, active form of PI3K and found that enhanced PI3K activity promoted keratinocyte proliferation, motility, and delayed differentiation (**Figure 5B**) (91). Further, PI3K/Akt pathway is likely to mediate TGF-β1-induced α-SMA expression and myofibroblast differentiation in dermal fibroblasts, indicating that PI3K/Akt pathway plays a crucial role in wound contraction (94). PI3K/Akt can also activate the Mammalian target of rapamycin (mTOR), and the PI3K/Akt/mTOR pathway was demonstrated to enhance inflammation, angiogenesis and deposition of ECM in HTS and dysregulation of the PI3K/Akt pathway in skin tissue gives rise to pathological outcomes characterized by excessive proliferation (90, 92). Tu et al. applied CUDC-907, a dual inhibitor of PI3K/Akt/mTOR pathway and histone deacetylases 2 (HDAC2) to human keloid fibroblasts (KFs), resulting in suppressed KF proliferation, migration, collagen production as well as reduced TGF-β1 *in vitro* (**Figure 5C**) (92). The CUDC-907 also attenuated collagen deposition and angiogenesis in the keloid xenograft mouse model (**Figure 5D**) (92). Therefore, PI3K/Akt pathway inhibitors would be good pharmacological candidates for stretch-induced HTS.

### Rho GTPases

**Figure 6:**
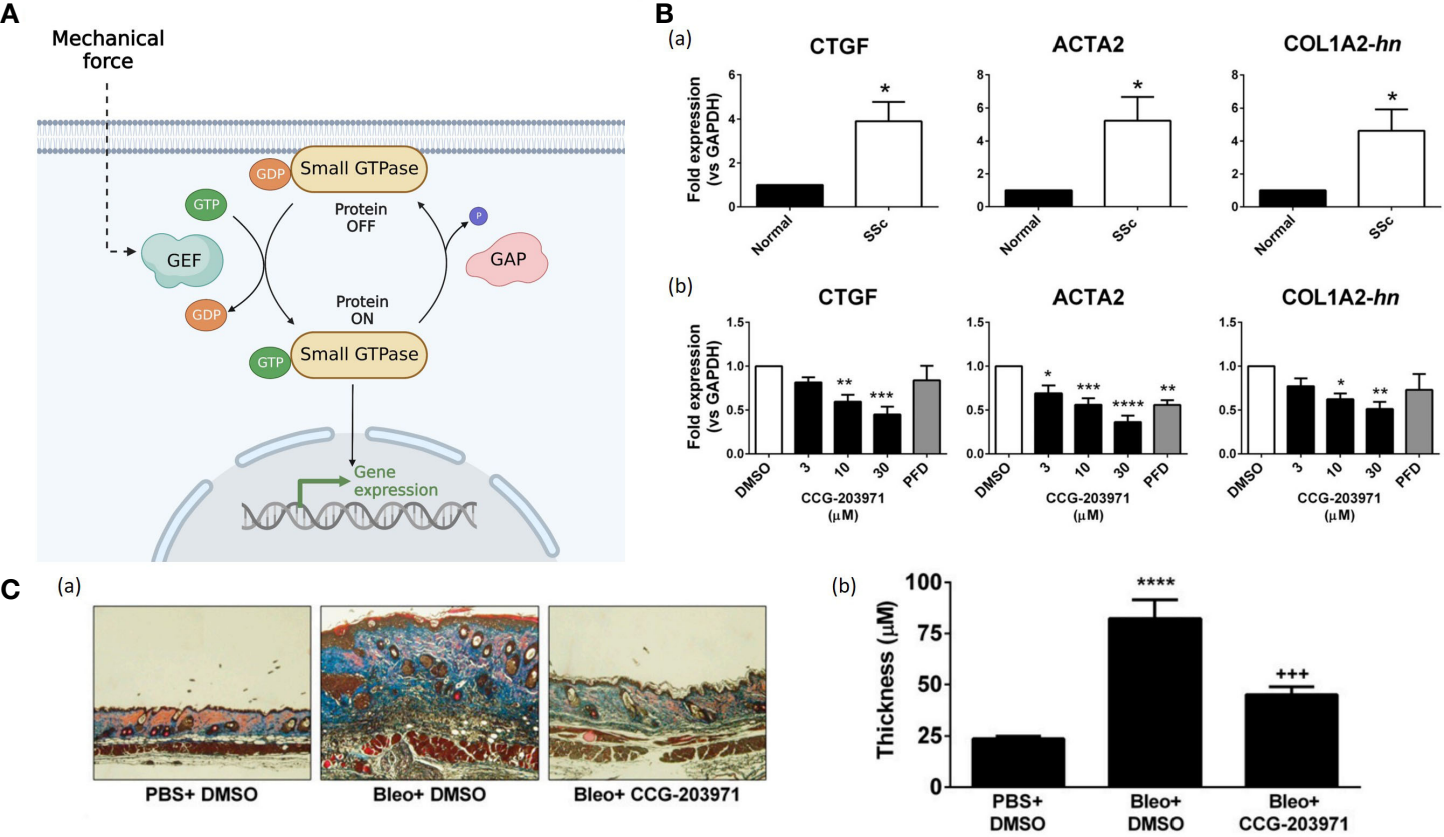
**(A)**. The small GTPases serve as molecular switches to regulate signal transduction pathways. The input induces GEFs activation, facilitating the conversion from inactive GDP-bounded configurations to active GTP-bound configurations. While GAPs mediate the hydrolysis of GTP, leading to the inactivation of the small GTPases. (Created with BioRender.com). **(B)** (a) The mRNA expression for connective tissue growth factor (CTGF), alpha smooth muscle actin (ACTA2), and collagen (COL1A2) are quantified by qPCR (96). All those fibrotic markers are up-regulated in SSc-patient dermal fibroblasts compared to normal human dermal fibroblasts (96). (b) CCG-203971 treatment reduces the expression of CTGF, ACTA2, and COL1A2. Before mRNA isolation, SSc dermal fibroblasts were treated for 24 hours in the presence of the indicated concentration (mM) of CCG-203971 or 300 mM PFD (96). Data are mean 6 S.E.M. of samples from at least four individuals. (*P<0.05, **P<0.01, ***P<0.001, ****P<0.0001 versus DMSO control.) (96) **(C)**. CCG-203971 prevents bleomycin-induced fibrosis *in vivo*. (***P<0.001, ****P<0.0001.) (96).

Rho GTPases are small GTPases belonging to the Ras superfamily. Approximately 20 members of the Rho GTPase family have been found in the human genome, including RhoA, Rac1 and Cdc42 (95). These GTPases serve as molecular switches by binding to guanosine triphosphate (GTP) and guanosine diphosphate (GDP). They are activated by Rho guanine nucleotide exchange factors (GEFs) and turned off by Rho GTPase activating proteins (GAPs) (**Figure 6A**) (97).

Rho GTPases regulate many cellular processes, including actin cytoskeleton remodeling, transcription, cell growth and proliferation, cell motility, morphology, and cell cycle progression (98). Mechanical loading evokes the reorganization of actin stress fibers and coordinates their orientation through Rho pathway (99). The best-characterized ones are RhoA and its downstream component, Rho-associated kinase (ROCK), which can generate contractile forces by mediating the functions of myosin II and actin filaments (100). Bond et al. found that the expressions of Rho and ROCK in scar fibroblasts markedly increased compared to that in surrounding normal tissue (101). After using Fasudil, a selective ROCK inhibitor, in rodent excisional wound models, the wound area became smaller and wound closure was delayed due to inhibition of fibroblast and myofibroblast contractility, indicating that ROCK inhibition might be potent prevention for scar contractures (101). Richardson et al. found that inhibition of Rho kinase or ROCK in adult zebrafish resulted in significantly slower re-epithelialization of full- and partial-thickness wounds (102). Besides, Haak et al. found that primary dermal fibroblasts from patients with systemic sclerosis (SSc) showed overexpression of myocardin-related transcription factor (MRTF)-and serum response factor (SRF)-regulated genes, which were highly correlated to the activation of Rho GTPase (**Figure 6B**) (96). Usage of CCG-203971 (the MRTF/SRF inhibitor) inhibited those fibrosis markers in SSc-patient dermal fibroblasts and attenuated skin-thickening and collagen deposition in a bleomycin-induced skin injury murine model (**Figures 6B, C**) (96). Hence, Rho inhibitors could be a potential therapeutic option to prevent wound fibrosis and contracture.

### Mechanosensitive ion channels

**Figure 7:**
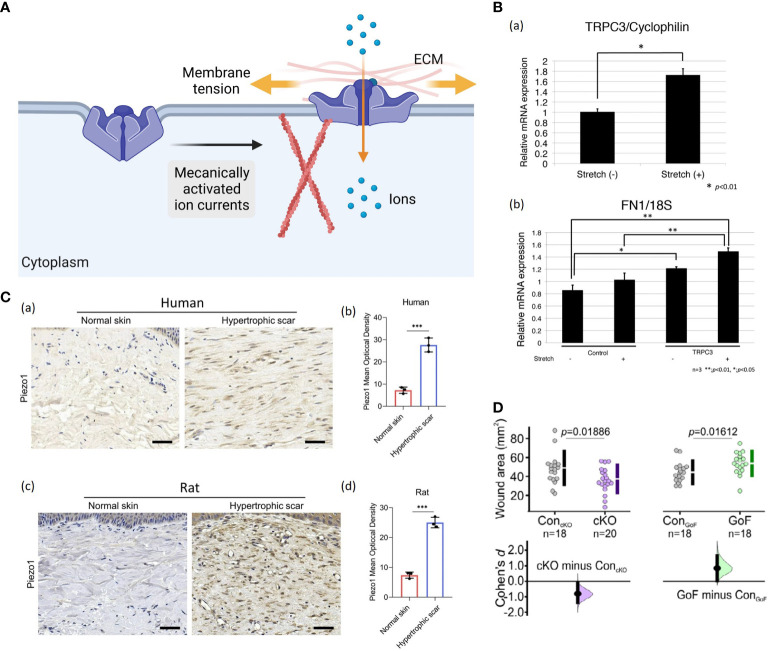
**(A)** A possible schematic of the ion channel. When membrane tension is low, the ion channel protein remains in a closed conformation (56). When membrane tension elevates by direct deformation of the lipid bilayer or indirectly by applying forces on the ECM and/or cytoskeleton, opening the ion channel protein allows inward currents (56). (Created with BioRender.com). **(B)** Increased TRPC3 channel expression and fibronectin production in stretched human cells: (a) The qRT-PCR demonstrated that stretched cells showed more TRPC3 mRNA expression compared to normal, unstretched fibroblasts. Data represent the means ± SD of 3 samples. *P < 0.01 (103). (b) Fibronectin production was increased in TRPC3 overexpressing fibroblasts when they were subjected to repetitive stretching. **P < 0.001, *P < 0.005 (103). **(C)** (a, b) Images and quantitative analysis of Piezo1 in human normal skin and HTS. (Scale bar = 50 μm) (104). (c, d) Images and quantitative analysis of immunohistochemistry staining of Piezo1 in rat normal skin and HS. (Scale bar = 50 μm) (104). The results are expressed as the means with SD (n = 3). The T-test is used for all analyses. ***P < 0.005. **(D)** The Piezo 1 knockout mice displayed significantly smaller wound areas compared to their control littermates, while Piezo1 gain-of-function mice showed larger wound areas, indicating that increased channel activity leads to impaired wound closure (105).

Ion channels are proteinaceous pores embedded in the plasma membrane, which can be activated by various physical or chemical stimuli, including mechanical forces (**Figure 7A**) (106).

Calcium influx *via* transient receptor potential (TRP) channels has been found to play a crucial role in response to mechanical input (107). TRP channels are divided into six subfamilies, including TRPC (canonical), TRPV (vanilloid), TRPM (melastatin), TRPA (ankyrin), TRPP (polycystin), and TRPML (mucolipin) (108). Several of them are potential mechanical force transducers that participate in HTS formation. Davis et al. confirmed that TRPC6-mediated calcium signaling induced fibroblast to myofibroblast transdifferentiation, which is indispensable for dermal wound healing (109). Ishisel et al. found that TRPC3 expression in the fibroblasts of stretched human scar tissue was increased, which induced calcium influx and ultimately led to scar contracture by upregulating fibronectin production (**Figure 7B**) (103). Ishii et al. showed that utilization of TRPV2 channel inhibitors effectively attenuated differentiation of dermal fibroblasts and contraction in rat model (110). In addition, Piezo proteins, including Piezo1 and Piezo2, have been identified as the members of mechanically activated cation channels (MACs) recently (111). He et al. demonstrated that Piezo1 was highly expressed in human and rat HTS tissues, especially in myofibroblasts (**Figure 7C**) (104). *In vitro*, cyclic mechanical stretch markedly triggered Piezo1 overexpression and Piezo1-dependent calcium influx in human dermal fibroblasts, leading to elevated cell proliferation, migration, differentiation and collagen production (104). Holt et al. found that epidermal-specific Piezo 1 knockout mice exhibited accelerated wound closure compared to gain-of-function mice (**Figure 7D**) (105). The *in vitro* experiment demonstrated that Piezo 1 activity induced retraction in keratinocytes during re-epithelialization, which caused delayed wound healing (105).

It’s becoming apparent that ion channels contribute to stretch-inducing HTS formation. However, investigations of those pathways are still rudimentary. Further studies are expected to reveal these mechanisms.

## Clinical and potential biological strategies for HTS prevention and treatment

HTS raises cosmetic problems, functional problems and patients’ subjective symptoms such as pruritus and pain, which dramatically affect patients’ physical status, psychological health and quality of life. However, the mechanism underlies HTS formation is complicated and yet to be elucidated. Hence current strategies hardly attain satisfactory outcomes. Advances in understanding mechanical forces and mechanotransduction highlight mechanical offloading and mechanomodulation for scar management. Here we introduce current clinical and potential biological strategies that conduct tensile reduction or mechanoregulation for HTS prevention and treatment.

### Tension-free sutures

Cutaneous injuries require thorough irrigation, removal of debris, proper selection of suture material and meticulous suture with minimal tension (112). Numerous techniques have been used to improve cosmetic outcomes in wound closure and scar revision, such as fascial tensile reduction sutures, Z-plasty, W-plasty, geometric broken line closure etc (113). The skin edges should contact in a slightly everted fashion to yield a less visible scar (114).

### Skin taping

Skin taping is a noninvasive scar management modality that can reduce skin tension and minimize scarring. The early application is routinely when the wounds are closed or after removal of sutures after surgical intervention, which shows good performance in holding the wound intact and isolating the incision from shear forces (115). The late application to established abnormal scars also indicates effectiveness. Scar volume, softness and color change can be notably improved (116). A reduction in pain and itch have also been found, subsequently increasing patient compliance (116). However, this strategy has potential complications such as superficial rash and demands high compliance.

### Silicone-based materials

Silicone-based materials are considered the first-line option for scar management, which should be used after the wound has fully epithelialized and until scar maturation (117). While the exact mechanisms of silicone-based products are yet to be fully agreed upon, their clinical effects remain undisputed (118). The silicone gel sheet reduces the tension along the border between wounded and normal skin by transferring tension from the wound bed to the lateral edge of the silicone gel sheet (119). The occlusion and hydration provided by silicone-based materials are also cited as a key mode of action (118, 120, 121). Besides, A prospective controlled clinical trial by Choil et al. demonstrated that early application of silicone gel sheet could down-regulated the expression of TGF-β1 and PDGF in both epidermis and dermis, which might mediate its clinical effect in scar prevention (122).

### Botulinum toxin type A

Botulinum toxin is a protein neurotoxin produced by the anaerobic spore-forming bacterium *Clostridium botulinum*. Intralesional injection of botulinum toxin type A (BTA) is an increasingly popular procedure in scar management. BTA can alleviate tension around the scar possibly in two ways: (1) temporarily paralyzing the underlying muscle, as well as (2) modulating fibroblast activity and reducing expression of TGF-β in dermis (123, 124). There is emerging evidence that early postoperative administration of BTA yields reduced scar, including cleft lip repair (125, 126), thyroidectomy (127), etc. BTA also demonstrates efficacy to established HTS (124). Besides, intralesional injections with BTA have improved clinical efficacy in treating HTS with lower pain compared to intralesional injections with corticosteroid (128). However, the usage of BTA has not been considered a conventional therapy for scar management. The injection protocol of BTA is varied among studies (129). A consensus on the injection time and BTA concentration are required before widespread clinical practice and further investigation.

### Microneedles

**Figure 8:**
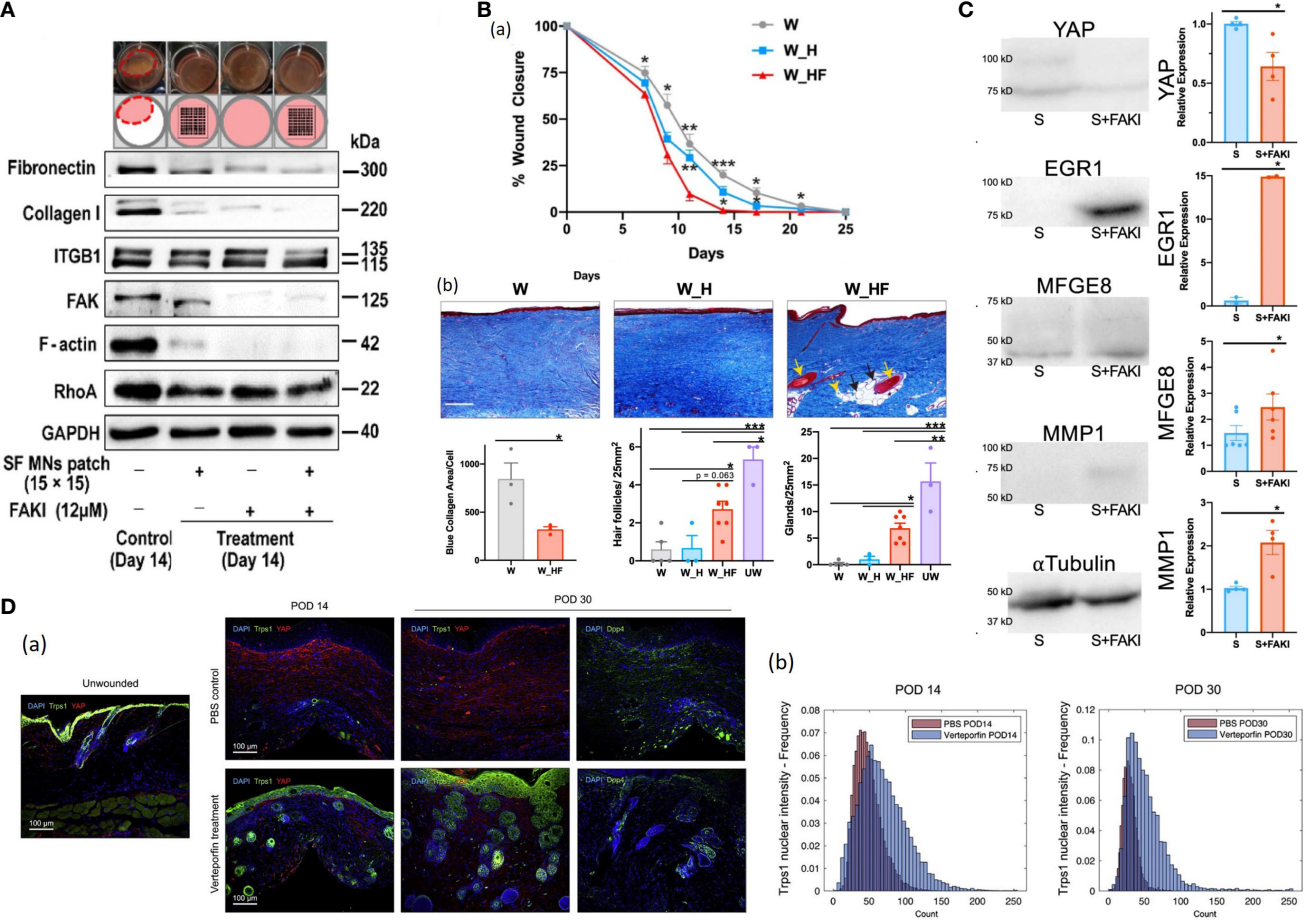
**(A)** Western blotting of integrin, FAK, RhoA, and F-actin assembly, as well as type I collagen and fibronectin involved in the ECM network in fibroblasts with the treatment of FAKI, SF MNs, and FAKI + SF MNs (130). **(B)** (a) Wounds treated with FAKI hydrogels (W_HF) show fully healed at postoperative day (POD) 14 ± 2.3, more than 10 days earlier than wounds treated with standard dressings (W) or empty hydrogels (W_H) *P < 0.05; **P < 0.01; ***P < 0.001 (131). (b) Masson’s Trichrome staining of healed scar to assess the presence of hair follicles (yellow solid arrows), secondary cutaneous glands (black solid arrows), and intradermal adipocytes proximal to the appendage structures (yellow dashed arrows) (131). Scale bar: 200 µm. Blinded experts counted the hair follicles (***P = 0.0005, *P = 0.021) and cutaneous glands (***P = 0.0001, **P = 0.0026, *P = 0.0445) (131). Collagen blue area quantified with custom MATLAB algorithm (*p = 0.0361) (131). **(C)** Mechanical strain induces fibrotic YAP mRNA and protein expression of human fibroblasts (131). FAK inhibition increased EGR1, MFGE8 and MMP1 expression while decreasing YAP expression (131). **(D)** (a) IF histology for YAP (red) and Trps1 (green; left) or Dpp4 (CD26; far right) in UW skin and PBS- or verteporfin-treated wounds at indicated time points (132). (b) Quantification of Trps1 nuclear localization by Biodock AI automated analysis (132).

Microneedles (MNs) are micron-sized devices extensively used in the cosmetic area. The microneedles have also been applied to minimize scar with characteristics of good biocompatibility, painlessness and minimal invasion. Zhang et al. demonstrated that a microneedle patch made of biocompatible silk fibroin could down-regulate scar formation by impeding the mechanical communication between ECM and fibroblasts and attenuating integrins-FAK mechanotransduction (**Figure 8A**) (130). In addition to the inherent therapeutic effect of microneedles, they can serve as transdermal drug carriers with site-specific delivery.

### Potential pharmacological strategies targeting mechanotransduction pathway

It has been identified that mechanotransduction is critically essential in skin wound healing outcomes, and several studies have uncovered that mechanotransduction pathways components can be potential therapeutic targets for HTS. For example, Chen et al. employed VS-6062 (a FAK inhibitor) hydrogels to the partial-thickness excisional wounds on the porcine dorsum. The treated wounds exhibited accelerated wound healing and skin regeneration with secondary structures (**Figure 8B**) (131). They found that the FAK-inhibited porcine and human wounded dermal fibroblasts reduced profibrotic transcriptional signatures and exhibited regenerative property characterized by EGR1, MMP1, and MFGE8 expression *via* Akt signaling (**Figure 8C**) (131). Furthermore, Chen et al. constructed a porcine model of autologous split-thickness skin grafting (STSG) and applied hydrogel dressing containing VS-6062 to those treated wounds (133). Blockade of FAK signaling up-regulated anti-inflammatory transcriptional profiles in myeloid cells during the early stage of healing and shifted transcriptional states of fibroblasts from profibrotic to regenerative at the late stage, ultimately leading to reduced scar contracture, promoted dermal remodeling and improved biomechanical skin properties (133). Mascharak et al. demonstrated that injection of verteporfin (an inhibitor of YAP) into mice dorsal incisions or fibroblast-specific transgenic YAP knockout yielded regenerative skin appendages, ultrastructure and mechanical strength instead of scarring (134). Their follow-up research revealed that YAP-inhibited fibroblasts upregulated Transcriptional Repressor GATA Biding 1 (Trps1) and activated Wnt signaling, implicating the mechanisms of YAP-inhibition-induced skin regeneration (132, 135)(**Figure 8D**). It’s noteworthy that those strategies are still under in the course of development and has not been carried out in humans. Collectively, those findings provide a promising direction for future efforts to develop therapies for HTS formation and other fibrosis.

## Conclusions

Despite decades of research, the cellular mechanisms governing cutaneous wound healing have only partially been revealed. A growing number of studies provide insight into the role of mechanotransduction signaling pathways in wound scar formation and skin regeneration. A comprehensive understanding of these regulatory networks will facilitate the development of novel therapeutics for HTS. Investigation in this field is still in its infancy. More studies are urgently needed to prevent, reduce, or even reverse scar formation ultimately.

## Author contributions

JY, SZ, CY, YW, NZ, and HH designed, wrote, and revised the manuscript. BS and QZ revised the manuscript. All authors contributed to the article and approved the submitted version.

## Funding

This work was supported by the Research and Develop Program, West China Hospital of Stomatology Sichuan University (RD-02-202107), Sichuan Province Science and Technology Support Program (2022NSFSC0743; 2022NSFSC1519), and the National Natural Science Foundation of China (81974147; 81800951).

## Conflict of interest

The authors declare that the research was conducted in the absence of any commercial or financial relationships that could be construed as a potential conflict of interest.

## Publisher’s note

All claims expressed in this article are solely those of the authors and do not necessarily represent those of their affiliated organizations, or those of the publisher, the editors and the reviewers. Any product that may be evaluated in this article, or claim that may be made by its manufacturer, is not guaranteed or endorsed by the publisher.
